# Magneto-optical detection of spin accumulation under the influence of mechanical rotation

**DOI:** 10.1038/s41598-018-20269-5

**Published:** 2018-01-31

**Authors:** Atsufumi Hirohata, Yuji Baba, Benedict A. Murphy, Benny Ng, Yunqi Yao, Kazuki Nagao, Jun-young Kim

**Affiliations:** 1 Department of Electronic Engineering, University of York, Heslington, York, YO10 5DD UK; 2 Department of Materials Science and Technology, Nagaoka University of Technology, Nagaoka, 940-2188 Japan; 3 Department of Physics, University of York, Heslington, York, YO10 5DD UK; 4 Department of Electronic Engineering, City University of Hong Kong, Kowloon Tong, Hong Kong; 5 Department of Electrical Electronics and Information Engineering, Nagaoka University of Technology, Nagaoka, 940-2188 Japan

## Abstract

The generation of spin-polarised carriers in a non-magnetic material holds the key to realise highly efficient spintronic devices. Recently, it has been shown that the large spin-orbit coupling can generate spin-polarised currents in noble metals such as tungsten and platinum. Especially, if small samples of such metals are rotated on a plane disc in the presence of a perpendicular magnetic field, the orbital angular momentum is altered leading to a segregation of spin up and spin down electrons, *i*.*e*., a spin current in the samples. This is manifested via an induced magnetic moment on the metal. In this letter, magneto-optical Kerr effect (MOKE) is used to detect induced magnetic moments which allows remote measurements on metal samples rotating at 100~210 Hz. Our results confirm the mechanical generation of spin-polarised currents via optical detection of spin accumulation.

## Introduction

Electron spin angular momentum is conserved in interactions with magnetic moments, circularly-polarised photons and electrons^1,2^. A steady flow of spin angular momentum in absence of any net charge flow is known as a pure spin current. This can be treated as a spin-polarised current where the charge components of the spin-up and down channels have opposite directions and cancel. The basis of spin currents and their manipulation lies in the control of the angular momentum of the electrons irrespective of their source. To date, spin currents have been generated predominantly by spin injection from a ferromagnet, application of electro-magnetic fields and Zeeman splitting^1,2^. Recently, thermal gradient^3^ and liquid-metal motion^4^ have been demonstrated to generate spin separation. Furthermore, generation of spin current in a non-magnetic metal by mechanical rotation has been proposed^5,6^ but has not yet been successfully achieved^7^ due to the presence of the Barnett effect^8^.

In 2011, Matsuo *et al*.^5^ proposed a method for generation of a pure spin current by mechanical rotation. By solving the Pauli-Schrödinger equation, they predicted that a spin current could be generated via angular momentum carried by a conductor rotating mechanically at high speed. The phenomenon arises from Einstein’s principle of equivalence for inertial and gravitational effects. Matsuo *et al*. derived the Pauli-Schrödinger equation in a rotating frame, where the electric field component was modified to have an additional contribution given by $(\overrightarrow{{\boldsymbol{\Omega }}}\times \overrightarrow{{\boldsymbol{R}}})\times \overrightarrow{{\boldsymbol{B}}}$ term, where $\overrightarrow{{\boldsymbol{\Omega }}}$ is angular frequency, $\overrightarrow{{\boldsymbol{R}}}$ is distance from the centre of rotation and $\overrightarrow{{\boldsymbol{B}}}$ is applied magnetic field. The additional term gives rise to spin-dependent wave packet velocities. For materials with a large spin-orbit coupling parameter such as tungsten (W) or platinum (Pt), a detectable spin imbalance is expected to accumulate at the edges of the sample as electrons with opposite spins migrate in opposite directions. This would lead to an apparent out-of-plane magnetic moment (*m*_*z*_) at the edge of the sample, the magnitude of which depends on the mechanically induced angular momentum.

**Figure 1:**
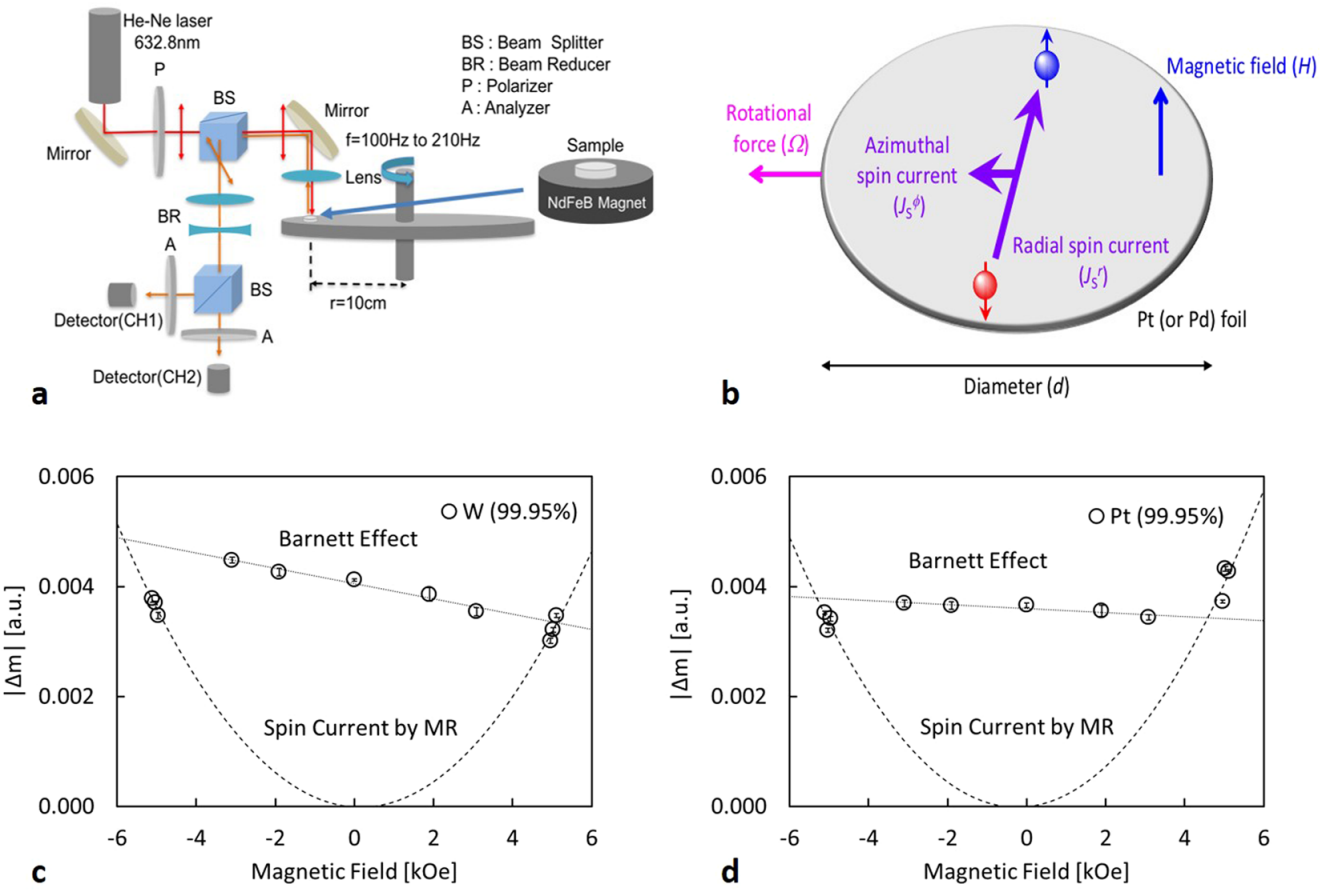
Magneto-optical measurement set-up and results. (**a**) Magneto-optical measurement set-up. The magnified diagram in (**b**) shows the distribution of the generated spin current. Magnetic field dependence of measured gradients of MOKE signals with frequency from (**c**), W and (**d**), Pt foils. Linear and parabolic fits represent contributions from the Barnett effect and mechanically induced spin currents, respectively.

The magnitude of the spin current generated by a uniformly rotating body is given by^6^

1 $$|\overrightarrow{{{\boldsymbol{J}}}_{{\bf{S}}}}|=2neR{\eta }_{{\rm{S}}{\rm{O}}}\frac{\hslash {\rm{\Omega }}}{{\varepsilon }_{{\rm{F}}}}{\omega }_{{\rm{C}}},$$

where $\overrightarrow{{{\boldsymbol{J}}}_{{\bf{S}}}}$ is the spin current density, *e* is the electron charge, *n* is the electron density, *R* is the radius of rotation, *η*_SO_ is the spin-orbit coupling parameter of the material, *Ω* is the angular frequency and *ε*_F_ is the Fermi energy. The term *ω*_C_ = *qB*/*m* is the cyclotron frequency for the wave packet of electrons treated in the derivation, where *q* is the charge of the wave packet and *m* is its mass. In the case of *B ≈* 1 T, *Ω ≈ 1* *kHz*, *η*_SO_ *≈* 0.59 (as in Pt), *k*_*F*_ *≈* 10^10^ m^−1^ and *R ≈* 0.1 m, Matsuo *et al*. estimates the spin current accumulated at the edges of the Pt foil to be of $|\overrightarrow{{{\boldsymbol{J}}}_{{\bf{S}}}}|$ ≈ 10^8^ A m^−2^. In the presence of impurity scattering, the spin current for the radial (${J}_{{\rm{S}}}^{r}$) and azimuthal (${J}_{{\rm{S}}}^{\varphi }$) directions (see Fig. 1b) is given by^6^

2 $${J}_{{\rm{S}}}^{r}=\frac{\tau {\omega }_{{\rm{C}}}}{1+{(\tau {\omega }_{{\rm{C}}})}^{2}}|\overrightarrow{{{\boldsymbol{J}}}_{{\bf{S}}}}|,$$

3 $${J}_{{\rm{S}}}^{\varphi }=\frac{{(\tau {\omega }_{{\rm{C}}})}^{2}}{1+{(\tau {\omega }_{{\rm{C}}})}^{2}}|\overrightarrow{{{\boldsymbol{J}}}_{{\bf{S}}}}|,$$

where *τ* is the relaxation time due to the impurity scattering. With our experimental condition, *τω*_C_ is expected to be ~10^−3^ for Pt, and the radial component will dominate over the azimuthal element. As schematically shown in Fig. 1(b), accumulated spins with opposite signs at both ends of the samples can be detected as an in-plane “magnetic moment” using our MOKE set-up. This is due to small misalignment of the incident and reflection beam from the plane normal of the sample in our setup. Hence, our measurement is sensitive to the mechanically-induced spin currents.

In this study, we developed a highly sensitive magneto-optical set-up to measure a mechanically-induced spin current in paramagnetic foils. We found that small magnetic fields below |*H*| =  ±4 kOe only induce the Barnett effect for the foil attached on a rotational plate with the radius of 0.10 m. On the other hand, fields above ±4 kOe were found to generate the spin current discussed above. Such a spin current proves that the spin-polarised carrier can be generated by mechanical rotation, which is analogous to the spin Hall effect.

## Results

Paramagnetic W and Pt foils (99.95% purity) were chosen due to their large values of ***η***_SO_ arising from high density of states at the Fermi level. Here, 100-µm-thick Pt and body-centred cubic W samples have opposite spin-Hall angles, 0.056 for Pt^9^ and −0.35 (−0.5) for W^10^ (W − Ox^11^). The foils of 2 mm diameter were polished to a <0.3 µm surface roughness. The samples were mounted on a NdFeB permanent magnet painted in matte black with surface stray fields between 1 kOe and 5 kOe. For each measurement the NdFeB magnet was fixed at a radial distance of 0.10 m on a balanced carbon fibre plate painted in matte black and rotated at frequencies of up to *f* = 210 Hz (12,600 rpm) thereby varying the angular momentum. The experimental arrangement is shown in Fig. 1a. The spin current in the foil (see Fig. 1b) was observed via the resulting induced moment using a magneto-optical Kerr effect (MOKE) magnetometer. It should be noted that the MOKE signals only represent the induced moments near the surface of the paramagnetic foils as their thicknesses, ~80 μm, well exceed the penetration depth of the He-Ne laser beam, <100 nm as detailed in sample preparation in Methods. A direct measurement of the change in moment was made by taking the difference between two signals registered by two photodetectors using a 3.5 GHz oscilloscope (Tektronix DPO7354C) (see Methods for details).

Figures 1c and d shows the variation of the gradient of the MOKE signals, |Δm|, due to the induced moments in W and Pt foils, respectively. The MOKE signals were measured as the difference in two photodetector signals with the rotational frequency between 150 and 210 Hz at a fixed radius of 0.10 m. Here, Δm is expected to be proportional to the spin current induced in the W and Pt foils. The details of the signal acquisition are described in Methods. The zero-field data were taken from W and Pt foils attached to a brass tube with the same dimensions with the permanent magnets used in this study. The output gradient is directly proportional to the sum of the fields from the magnet and the induced magnetic moment due to the Barnett effect below |*H*| =  ± 4 kOe. Here, the magnetic flux density from the Barnett effect (*B*_B_) is induced perpendicular to the foil by the rotation of electrons in the sample^8^:

4 $${B}_{{\rm{B}}}=2\frac{m}{e}{\rm{\Omega }}{\rm{.}}$$

By considering the average angular frequency used in our measurements (150 Hz), *B*_B_ is estimated to be 1.71 × 10^−9^ Wb m^−2^. This gives a magnetic moment of 5.40 × 10^−15^ Wb within the 2-mm-diameter circular foil, indicating that our MOKE set-up is sensitive to such a small magnetic moment. Using the least squares method, these results can be fitted to parabolic and linear functions as detailed in Discussion.

## Discussion

The gradient of our MOKE signal with the rotation frequency is expected to contain information from three different phenomena: reduction of the laser beam exposure time due to faster rotation, moment induced from the Barnett effect, and spin accumulation from the mechanically-generated spin current. First two phenomena, namely from the faster rotation and the Barnett effect, are expected to contribute linearly with the magnetic field strengths. On the other hands, the mechanically-generated spin currents have quadratic dependence on the field strength. As seen in Fig. 1(c) and (d), we compare the gradients of normalised MOKE signals from samples with different magnet field strengths in order to differentiate the effects of the mechanically-generated spin current from the first two contributions.

For |*H*| < 4 kOe, the MOKE gradient signal is linearly proportional to the field, as expected from the moments induced by the Barnett effect. The linear fitting of the data points within this field range results in the linear coefficients of (1.34 ± 0.19) × 10^−4^ and (4.2 ± 1.2) × 10^−5^ for W and Pt, respectively, giving the ratio of 3.2 ± 1.0. Furthermore, in order to quantify the moments induced by the Barnett effect, magnetic susceptibility values of our W and Pt foils were measured by VSM to be 3.0 × 10^−6^ and 2.6 × 10^−6^ (emu cm^−3^) Oe^−1^, respectively (see Methods for details), leading to the ratio of 1.2. We expect the discrepancy between the two ratios to be due to the errors in the estimation of the small foil volumes.

When |*H*| >4 kOe, the MOKE gradient signals start to show higher order field dependencies. This suggests the emergence of the mechanical spin current and the resulting magnetic moment^5^. The larger second-order coefficient of the W data, as compared to the Pt, agrees with the prediction as discussed in Eq. (2). The second-order field dependence of the induced moment is interpreted as the first experimental observation of mechanically induced spin currents. For our experimental condition for Pt, a (radial) spin current density of ≈3 × 10^3^ A m^−2^ should be induced according to Eq. (2). Matsuo *et al*.^6^, estimated the spin accumulation due to the mechanically-generated spin current to be around 0.05 neV for a Pt film with a length of 100 nm. Assuming that the magnitude of the spin accumulation is linearly proportional to the length of the film, the spin accumulation at the edges of our 2 mm diameter Pt film is estimated to be around 1 µeV. It should be noted that the mechanically-generated spin current is an order of magnitude smaller than the conventional spin current generated by spin pumping^12^, but the magnitude can be increased by using a larger rotational diameter and a higher angular frequency.

## Methods

### Sample preparation

W and Pt foils with thickness of 100 µm (99.95% purity) were glued onto NdFeB magnets and polished using diamond lapping pads (30, 15, 3 and 1 µm) to a 1 µm finish. The foils were then polished using water solution with aluminium oxide particles of 0.3 µm diameter. During polishing the samples were kept level on a tripod polishing mount. The thickness of the polished foils were measured to be (81.5 ± 1.6) µm and (79.5 ± 0.5) µm for W and Pt, respectively.

**Figure 2:**
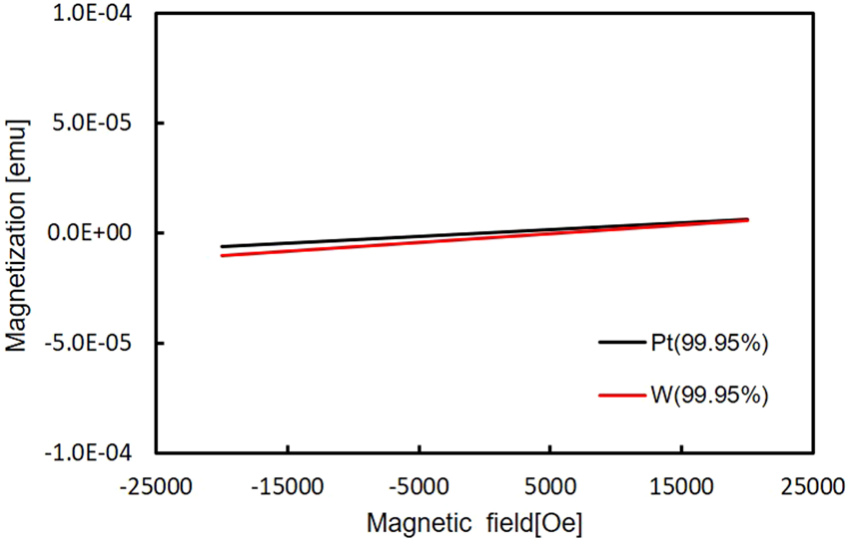
Magnetisation curves for the W and Pt foils. Magnetic field dependence of magnetisation for the W and Pt foils. The background signal due to the sample holder has been subtracted.

The magnetic susceptibility of these foils, *χ*, were then measured using a vibrating sample magnetometer (VSM, ADE, Model 10) at room temperature (see Fig. 2). The volume of the foils were measured by an optical microscope, which gave *χ* = 3.0 × 10^−6^ and 2.6 × 10^−6^ (emu/cm^3^) Oe^−1^ for W and Pt, respectively.

### Permanent magnets

**Figure 3:**
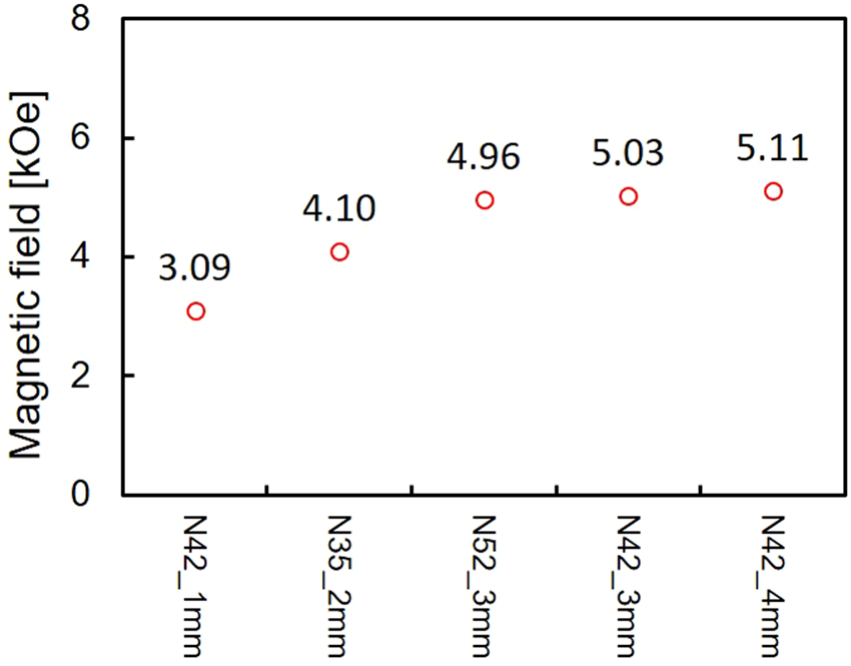
Measured magnetic fields generated by permanent magnets. Magnetic fields measured by a gaussmeter at room temperature.

Five different NdFeB permanent magnets were used for the measurements. All magnets were 5 mm in diameter with different thickness (1~4 mm) and were plated by Ni/Cu/Ni. The surface stray fields of these magnets were measured by a gaussmeter as shown in Fig. 3. A 5-mm-diameter brass piece was also used as a zero-field reference.

### MOKE set-up

**Figure 4:**
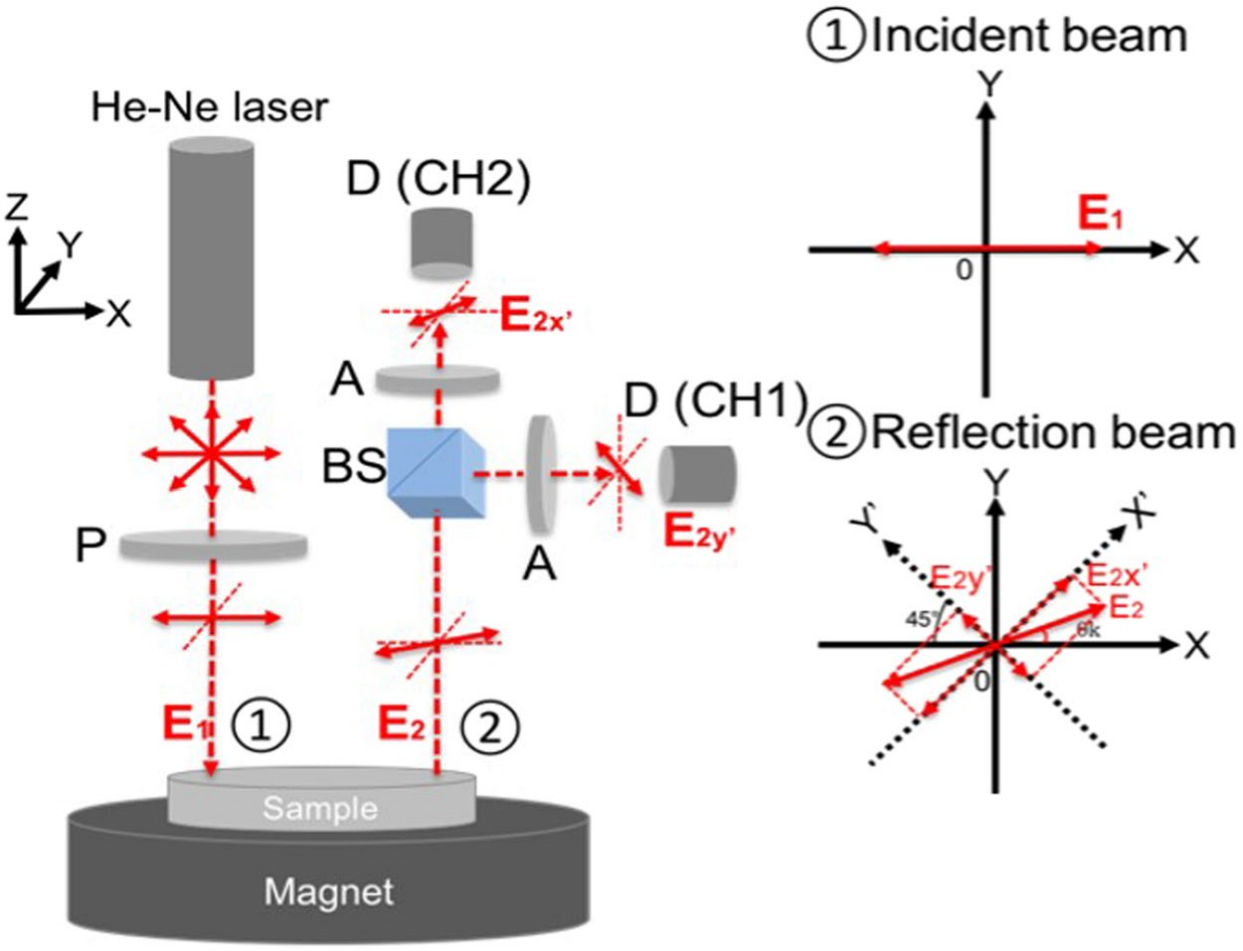
MOKE setup. Schematic diagram of the MOKE measurement system with the corresponding polarisation of incident and reflection beams.

He-Ne laser (Melles Griot, 05-STP-903, wavelength: 632.8 nm and power: 1.0 mW) was used to detect Kerr rotation of the linear polarisation of the light reflected from the surface of the paramagnetic foils. The rotation was caused due to the magnetic moment induced by a spin-polarised current accumulated at the edge of the foils. The beam was introduced in the plane normal to the surface of the sample centre with the typical penetration depth of <100 nm. As shown in Fig. 4, the reflected beam was split into two linearly polarised beams, and measured using two photodetectors (Thorlabs, DET36A Si Detector), CH1 and CH2, in the cross-Nicol configuration^13,14^. For demonstration, we assume that the incident beam is linearly polarised along the *x* direction:

5 $$\mathop{{{\boldsymbol{E}}}_{1}}\limits^{\longrightarrow}={E}_{0}(\begin{array}{c}1\\ 0\end{array}).$$

Upon reflection off the sample, the linear polarisation of the beam is rotated by the Kerr angle *θ*_*K*_:

6 $$\mathop{{{\boldsymbol{E}}}_{2}}\limits^{\longrightarrow}={E}_{0}(\begin{array}{cc}\cos \,{\theta }_{{\rm{K}}} & -\,\sin \,{\theta }_{{\rm{K}}}\\ \sin \,{\theta }_{{\rm{K}}} & \cos \,{\theta }_{{\rm{K}}}\end{array})(\begin{array}{c}1\\ 0\end{array})={E}_{0}(\begin{array}{c}\cos \,{\theta }_{{\rm{K}}}\\ \sin \,{\theta }_{{\rm{K}}}\end{array}).$$

The reflected beam then passes through a non-polarising beam splitter, where each-split beam passes through the linear polarisers set orthogonally at 45° and 135° from the *x*-axis, respectively, then reaches the two photodetectors CH1 and CH2:

7 $$\mathop{{{\boldsymbol{E}}}_{2,CH1}}\limits^{\longrightarrow}={E}_{0}\,\cos (\frac{\pi }{4}-{\theta }_{K})=\frac{{E}_{0}}{\sqrt{2}}(\cos \,{\theta }_{K}+\,\sin \,{\theta }_{K}),$$

8 $$\mathop{{{\boldsymbol{E}}}_{2,CH2}}\limits^{\longrightarrow}={E}_{0}\,\sin (\frac{\pi }{4}-{\theta }_{K})=\frac{{E}_{0}}{\sqrt{2}}(\cos \,{\theta }_{K}-\,\sin \,{\theta }_{K}).$$

The difference between the two photodetector voltages can be computed as follows:

9 $$\begin{array}{rcl}V1-V2 & = & |\overrightarrow{<mml:mpadded xmlns:xlink="http://www.w3.org/1999/xlink" voffset="0">{{\boldsymbol{E}}}_{2,CH1}</mml:mpadded>}{|}^{2}-|\overrightarrow{<mml:mpadded xmlns:xlink="http://www.w3.org/1999/xlink" voffset="0">{{\boldsymbol{E}}}_{2,CH2}</mml:mpadded>}{|}^{2}\\  & = & \frac{{{E}_{0}}^{2}}{2}[{(\cos {\theta }_{K}+\sin {\theta }_{K})}^{2}-{(\cos {\theta }_{K}-\sin {\theta }_{K})}^{2}]\\  & = & 2{{E}_{0}}^{2}\,\cos \,{\theta }_{K}\,\sin \,{\theta }_{K}={{E}_{0}}^{2}\,\sin \,2{\theta }_{K}\end{array}$$

For small Kerr rotation angles, $\sin \,2{\theta }_{{\rm{K}}}$ can be treated as $2{\theta }_{{\rm{K}}}$:

10 $${\rm{V1}}-{\rm{V2}}={\rm{2}}\,{E}_{0}^{2}{\theta }_{{\rm{K}}}\quad \quad {\rm{for}}\,{\rm{small}}\,{\theta }_{{\rm{K}}}.$$

Therefore, *θ*_K_ is proportional to the difference in the two photodetector voltages V1 − V2.

**Figure 5:**
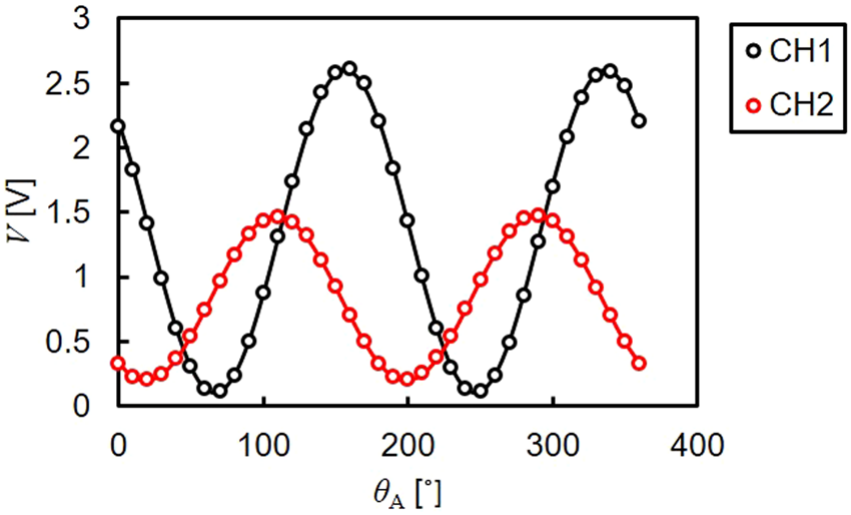
Calibration results. Polariser angle dependence of the detector signals. The data was taken at 10° steps and was fitted with a cos^2^*θ* function.

The cross-Nicol configuration was determined by calibrating the detector signals for every sample. Here, the polariser angle for the incident beam was fixed to be 0°, while the two analyser angles were selected to maximise the changes in the signals, i.e. at the point where the gradient of the sine MOKE signals takes the maximum, as shown in Fig. 5. For the calibration, the foil was rotated at a frequency of 20 Hz and the reflection was measured by the two detectors simultaneously with changing their polariser angles from 0 to 360° at steps of 10°. Representative results are shown in Fig. 5 for the Pt foil with the N42_4mm magnet (with the surface stray field of ~5.1 kOe). The detector polariser was then adjusted to maximise the gradient of the detector signals. A list of polariser angles used is shown in Tables 1 and 2.

**Table 1:** List of detecting polariser angles for the W foils.

Grade	Magnet		Analyser angle	
	Thickness [mm]	Direction	CH1 [°]	CH2 [°]
N42	4	−	20.6	69.5
N42	3	−	34.9	39.1
N52	3	−	25.4	50.3
N35	2	−	15.9	61.1
N42	1	−	18.3	71.1
Brass 2 mm			32.9	33.4
N42	1	+	38.9	31.1
N35	2	+	51.1	25.8
N52	3	+	20.8	68.1
N42	3	+	21.3	67.5
N42	4	+	27.2	50.3

**Table 2:** List of detecting polariser angles for the Pt foils.

Grade	Magnet		Analyser angle	
	Thickness [mm]	Direction	CH1 [°]	CH2 [°]
N42	4	−	21.5	67.6
N42	3	−	26.5	53.7
N52	3	−	20.2	71.0
N35	2	−	20.4	69.2
N42	1	−	20.8	62.1
Brass 2 mm			20.9	62.4
N42	1	+	19.8	65.8
N35	2	+	23.7	56.6
N52	3	+	21.1	63.7
N42	3	+	21.3	69.5
N42	4	+	21.5	63.9

### Signal Acquisition

**Figure 6:**
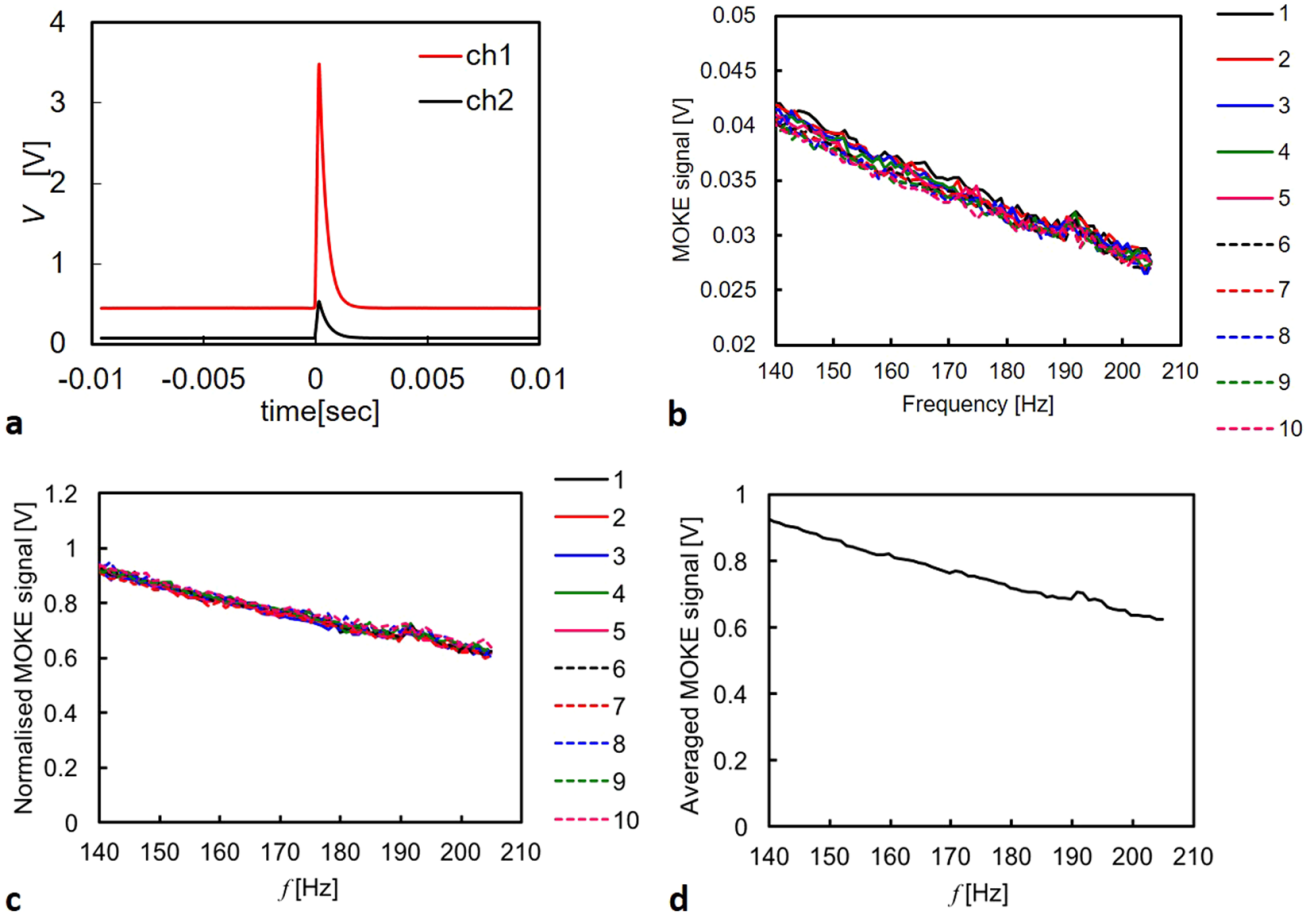
Signal acquisition. (**a**) Representative MOKE signals measured at the rotation frequency of 30 Hz. (**b)** Peak MOKE signals at the frequency between 100 and 210 Hz for the Pt foil with the magnet N42_4mm (~5.1 kOe). (**c)** Corresponding MOKE signals normalised to the signals measured at 100 Hz. (**d)** Averaged MOKE signals over 10 measurements shown in (**c)**.

Our MOKE signals were acquired at the rotation frequency between 100 Hz and 200 Hz. The frequency range was chosen so that the rotation of the disc was free from the resonance of neighbouring equipment and the sample plane became consistently perpendicular to the incident laser beam. As the sample only stayed under the laser beam for a short time (approximately 32 µs at 100 Hz) for each rotation, our signal was of a pulsed nature, as displayed in Fig. 6(a). Relatively long (~1 ms) pulse-widths of our signal, as compared to the estimated beam exposure time, were attributed to be due to long “cool-down” times of the CCDs in our photodetectors. The 3.5 GHz Tektronix oscilloscope was used to record our pulsed signal from the photodetectors, where a built-in program was used to extract the effective magnitude of each pulse peak as compared to the background level.

The measured peak magnitude value was then recorded against the rotation frequency, as shown in Fig. 6(b). The almost linear decrease of the peak height with the rotation frequency was due to the reduction in the time the sample stays under the laser beam as expected, leading to the drop in the measured intensity. In order to compensate for the slight changes in the optics alignment for each measurement, the obtained peak value was normalised to its value at 100 Hz, as seen in Fig. 6(c) and (d), so that the relative decrease in the signal with the frequency for different measurements could be compared quantitatively.

Without this normalisation step, the peak height values from different measurements could not be compared directly due to the changes in the observed intensity from the non-identical optics alignment. We had tried to position the sample manually under the laser beam and measure the MOKE signal directly without rotation. However, the exact position of the beam relative to the sample was found to change each time we change the magnet or sample, which led to large and unsystematic changes in the measured MOKE signal. This was predominantly because the sample attached on a permanent magnet needs to be fixed on the rotating plate using glue, causing minor misalignment in the sample plane with respect to the rotating plate plane. Consequently, rotation of the sample was necessary, not only to investigate the rotational effects, but also for the normalisation of the measured signals. Gradients of the normalised signals were compared to quantify effects of spin currents from the rotation motion, as discussed in Introduction.

In summary, we have demonstrated experimentally non-linear dependence of the spin accumulation with field at the edge of paramagnetic foils on a rotating plate, as theoretically predicted^5^. This finding establishes an alternative method to generate a pure spin current induced by the conservation of angular momentum of electron spin under mechanical rotation. Our optical detection opens a new research field of spin mechatronics, which may offer a template to investigate further relationships between mechanical motion and spin angular momentum.

After submitting this manuscript, Kobayashi *et al*.^15^ reported a noble method of detecting spin currents generated by using Rayleigh-type surface acoustic wave (SAW) in Ni_81_Fe_19_/Cu bilayer system. By measuring microwave absorption at SAW excitation frequency at 1.5 GHz, a large spin-rotation coupling effect was observed.

## Electronic supplementary material

Supplementary Information
