# Supplementary material for: Magneto-optical detection of spin accumulation under the influence of mechanical rotation
Source: Sci Rep. 2018 Jan 31;8:1974. doi: 10.1038/s41598-018-20269-5 (PMC5792645; doi:10.1038/s41598-018-20269-5)
Supplement: Supplementary file 1 — Supplementary Information [file 41598_2018_20269_MOESM1_ESM.doc]

**Magneto-optical detection of spin accumulation under the influence of mechanical rotation**

Atsufumi Hirohata,^1,*^ Yuji Baba,^2^ Benedict A. Murphy,^3^ Benny Ng,^4^ Yunqi Yao,^4^ Kazuki Nagao ^5^ and Jun-young Kim ^3^

^1^ *Department of Electronics, University of York, Heslington, York YO10 5DD, U.K.*

^2^ *Department of Materials Science and Technology, Nagaoka University of Technology, Nagaoka 940-2188, Japan*

^3^ *Department of Physics, University of York, York YO10 5DD, U.K.*

^4^ *Department of Electronic Engineering, City University of Hong Kong, Kowloon Tong, Hong Kong*

^5^ *Department of Electrical Electronics and Information Engineering, Nagaoka University of Technology, Nagaoka 940-2188, Japan*

^*^ e-mail: atsufumi.hirohata@york.ac.uk

**Supplementary information**

**Error Analysis.** The measurement procedure generated a sequence of pulses depending on the frequency of rotation. Each data point was repeated between 3 and 7 times. A smooth curve was then fitted to the entire data set using an inverse cubic least squares fit. The final values of ΔV were obtained by subtracting the values of the curve for the magnet alone from that for the magnet and sample. The error in the final value was taken as the mean deviation of all measured points from the curve and the final error bars as the root of the average errors in quadrature. *i.e.*,


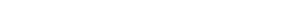
 (S1)
